# Association of endoscopic and CT response with pathological response after preoperative FLOT-based therapy for gastric and gastroesophageal junction cancer: a retrospective cohort study

**DOI:** 10.3389/fonc.2026.1960800

**Published:** 2026-10-16

**Authors:** Huseyin Gencer, Gokhan Ucar, Efnan Algın, Mehmet Ali Nahit Sendur, Erdal Birol Bostanci

**Affiliations:** 1 Department of Medical Oncology, Ankara Bilkent City Hospital, Ankara, Türkiye; 2 Department of Gastroenterological Surgery, Ankara Bilkent City Hospital, Ankara, Türkiye

**Keywords:** computed tomography, endoscopy, flot, gastric cancer, gastroesophageal junction cancer, pathological response, tumor regression grade

## Abstract

**Background:**

Pathological response to preoperative chemotherapy is prognostically relevant in gastric cancer, but reliable estimation before surgery remains challenging. We evaluated the relationship between routine endoscopic and computed tomography (CT) response assessments and major pathological response (MPR) after FLOT-based therapy.

**Methods:**

In this retrospective, single-center cohort study, 450 institutional records were screened and 47 patients with gastric or gastroesophageal junction cancer who underwent gastrectomy after FLOT-based therapy and had evaluable pre- and post-treatment endoscopy were included; 37 also had comparable paired CT assessments. Endoscopic and CT responses were classified as complete, partial, stable, or progressive. Pathology reports were harmonized into complete, near-complete, partial, and poor/no response, and MPR was defined as complete or near-complete response. Discrimination was assessed by receiver operating characteristic analysis, and paired endoscopy and CT areas under the curve (AUCs) were compared using the DeLong test. The combined endoscopy-plus-CT model was exploratory.

**Results:**

MPR occurred in 17 of 47 patients (36.2%), including pathological complete response (pCR) in 10 (21.3%). In the full cohort, endoscopy had an AUC of 0.619 (95% CI, 0.466-0.771) for MPR. In the paired cohort, AUCs were 0.561 (95% CI, 0.379-0.743) for endoscopy and 0.675 (95% CI, 0.501-0.848) for CT (difference, 0.114; p=0.35). The combined model yielded an apparent AUC of 0.692 (95% CI, 0.511-0.873). Both patients with endoscopic complete response had pCR, although this category identified only 2 of 10 pCRs.

**Conclusions:**

Routine endoscopic response showed limited discrimination for MPR. CT had a numerically higher AUC, but the paired comparison was inconclusive, and combining the modalities did not materially improve discrimination. Given the small paired cohort and heterogeneous pathology reporting, these findings are hypothesis-generating. Standardized prospective assessment is required before endoscopic or CT response can be used to estimate pathological response with clinical confidence.

## 1 Introduction

Gastric cancer remains a major cause of cancer-related morbidity and mortality worldwide (1, 2). For patients with resectable, locally advanced gastric or gastroesophageal junction (GEJ) adenocarcinoma, perioperative chemotherapy improves outcomes compared with surgery alone (3, 4). Following the FLOT4-AIO trial, fluorouracil, leucovorin, oxaliplatin, and docetaxel (FLOT) became a standard perioperative option for medically fit patients (5, 6).

The extent of pathological regression after preoperative therapy provides important prognostic information (7–10). However, pathological response can be determined definitively only after examination of the resection specimen. A reliable preoperative estimate could improve prognostic counseling, support treatment monitoring, and inform the design of future response-adapted studies.

Preoperative response assessment is difficult because endoscopy, CT, and surgical pathology evaluate different aspects of disease. Endoscopy directly visualizes the luminal and mucosal surface, whereas viable tumor may persist within deeper layers of the gastric wall. CT provides extraluminal and nodal information, but the primary gastric lesion is frequently non-measurable under RECIST v1.1, and response may therefore be determined by lymph nodes or other target lesions (11). Surgical pathology, in contrast, assesses treatment effect throughout the resected primary-tumor bed. Discordance among these modalities is therefore biologically and methodologically plausible.

Previous studies have reported associations between endoscopic response, pathological regression, and survival, but treatment regimens, assessment schedules, response definitions, and review procedures have varied substantially (12–16). Data from contemporary FLOT-treated surgical cohorts, particularly when response is assessed from routine clinical reports, remain limited.

We therefore evaluated the association and discriminative ability of routine endoscopic and CT response categories for MPR after preoperative FLOT-based therapy. We also explored whether combining the two modalities improved discrimination and examined the relationship between endoscopic complete response and pCR.

## 2 Materials and methods

### 2.1 Study design and cohort assembly

This retrospective, single-center cohort study was conducted at Ankara Bilkent City Hospital. Adults with gastric or GEJ cancer diagnosed between 16 July 2020 and 5 November 2025 were eligible if they received preoperative FLOT-based therapy followed by gastrectomy. Clinical TNM categories were analyzed as recorded in the study database. The manuscript was prepared with reference to STROBE and relevant STARD reporting items (17, 18).

The institutional screening file contained 450 records. Inclusion in the endoscopy-pathology cohort required (1) pretreatment and post-treatment/preoperative endoscopy, (2) sufficient lesion measurements and/or morphological description to classify response of the index lesion, and (3) an assessable gastrectomy pathology report with a documented tumor regression grade or pathological response. Records were excluded hierarchically in the order listed. A total of 403 records were excluded: no FLOT-based therapy (n=10), no post-treatment endoscopy (n=254), insufficient endoscopy report to classify response (n=122), and no tumor regression grade or pathological response documented in the pathology report (n=17). Forty-seven patients met all criteria and formed the endoscopy-pathology cohort. Comparable paired CT response was available in 37 of the 47 included patients, who formed the head-to-head cohort; the remaining 10 lacked comparable paired CT (**Figure 1**).

**Figure 1:**
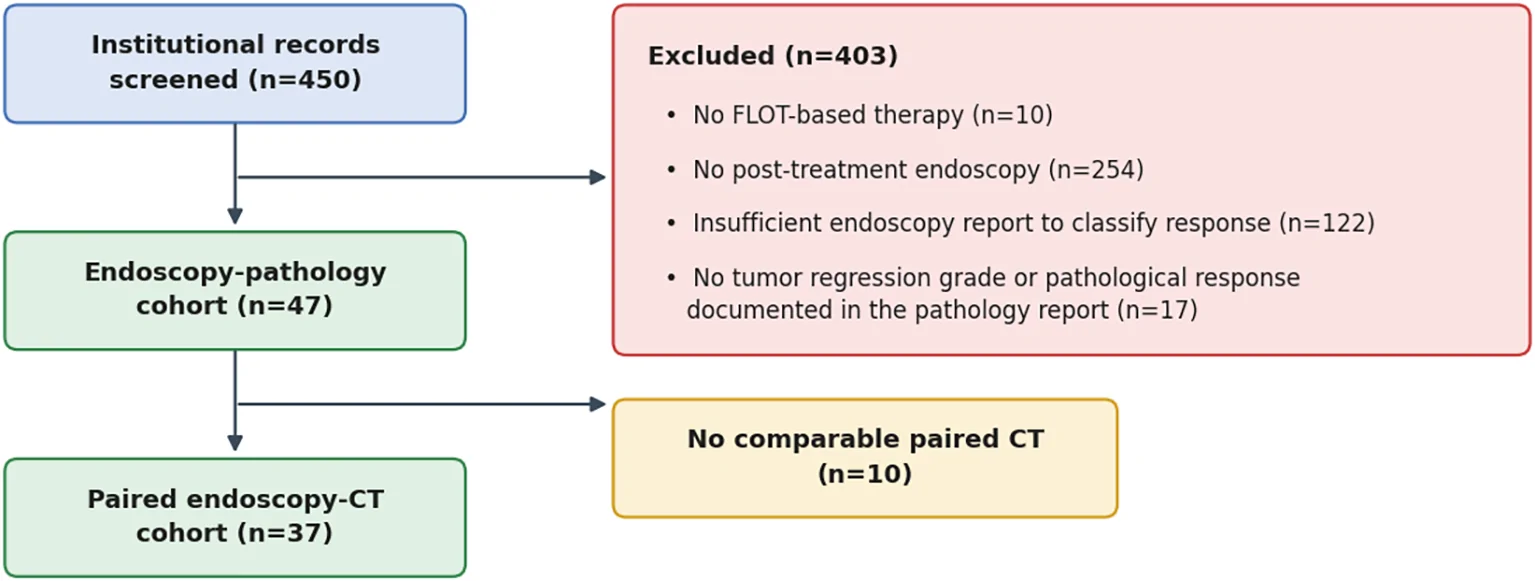
Study flow. Of 450 records screened, 403 were excluded: no FLOT-based therapy (n=10), no post-treatment endoscopy (n=254), insufficient endoscopy report to classify response (n=122), and no tumor regression grade or pathological response documented in the pathology report (n=17). Forty-seven patients were included in the endoscopy-pathology cohort, of whom 37 had comparable paired CT and formed the paired endoscopy-CT cohort. Exclusion reasons were applied hierarchically; each record is counted once under the first applicable reason.

Baseline clinical characteristics of the 403 excluded records were not abstracted into the study database, because the exclusion criteria were applied at the record-screening stage; a formal comparison of included and excluded patients was therefore not possible. Within the included cohort, patients with and without comparable paired CT were compared with respect to MPR prevalence and endoscopic response distribution (Section 3.3).

Demographic, clinicopathological, treatment, biomarker, response, and surgical variables were obtained from the study database.

### 2.2 Endoscopic and CT response assessment

Endoscopic response was classified as complete response (eCR), partial response (ePR), stable disease (eSD), or progressive disease (ePD) according to a predefined chart-review framework informed by Japanese gastric-cancer response conventions (19). For each patient, the index lesion was the primary gastric or GEJ tumor described in the pretreatment endoscopy report. The pretreatment and post-treatment/preoperative reports were reviewed together, and the following operational rules were applied in order: eCR, no visible tumor at the index site (scar, flat mucosal change, or healed ulcer only) and no new lesion; ePD, unequivocal enlargement of the index lesion, new tumor or new luminal stenosis attributable to tumor progression, or worsening obstruction; ePR, clear regression of the index lesion without meeting eCR criteria, defined as a reduction of at least 50% in the largest reported dimension when measurements were available or, when only morphological description was available, an explicit description of marked shrinkage, flattening of the tumor, or healing of the tumoral ulcer to a stage without residual mass; eSD, a lesion that was still visible without meeting the criteria for ePR or ePD. Reports lacking either a measurement or a morphological description sufficient to apply these rules were classified as non-assessable and the patient was excluded (n=122; **Figure 1**). For binary analyses, eCR and ePR were categorized as endoscopic response, whereas eSD and ePD were categorized as non-response.

CT response was classified as complete response (CR), partial response (PR), stable disease (SD), or progressive disease (PD) using a framework based on RECIST v1.1 (11). RECIST v1.1 was chosen because the aim of the study was to examine how response categories generated in routine clinical practice relate to pathological response, and RECIST provides standardized, predefined categories that are used for radiological reassessment in this setting and allow comparison across studies. We acknowledge that the primary gastric lesion is not always measurable under RECIST and that the radiological category may reflect changes in lymph nodes or other target lesions, whereas the pathological reference standard reflects treatment effect in the resected primary-tumor bed. This difference was anticipated and is considered in the interpretation of discordance between modalities (see Discussion). The CT category was taken from the radiology report when the report stated an explicit RECIST-based response. When the report gave lesion measurements without an explicit category, the category was derived from the reported measurements using RECIST v1.1 thresholds (CR, disappearance of all target lesions and of pathological lymph nodes; PR, at least a 30% decrease in the sum of diameters; PD, at least a 20% increase and an absolute increase of at least 5 mm in the sum of diameters or any new lesion; SD, neither PR nor PD). When the primary lesion was non-measurable, the category reflected the measurable target lesions (most often lymph nodes) and non-target assessment recorded in the report. Patients whose pre- and post-treatment CT examinations were not comparable (different protocol, missing baseline or restaging study, or no lesion description permitting classification) were considered to have no comparable paired CT (n=10). CR and PR were categorized as CT response, and SD and PD as CT non-response. CT analyses were restricted to the 37 patients with comparable pre- and post-treatment assessments.

Response classifications were based on routine clinical reports. No blinded central re-review was performed, and interobserver agreement could not be assessed. The available analysis dataset did not include the original images, raw lesion measurements, target-lesion definitions, or assessor characteristics.

### 2.3 Pathological reference standard

Treatment-response terminology in gastrectomy pathology reports was heterogeneous. Although the study protocol had planned to evaluate Becker or Mandard grading, no single system was used consistently in routine pathology reports. Ryan or modified Ryan grading was explicitly reported in 10 cases, Becker grading in 2, and a TNM8-based regression score in 2; 21 reports used an unnamed numerical score or grade, and 12 used descriptive terminology alone. Residual viable-tumor percentages were not consistently available, and no central slide review was performed.

To permit a common analysis, pathological response was harmonized into four ordered categories using terminology analogous to contemporary College of American Pathologists reporting: complete response (score 0), near-complete response (score 1), partial response (score 2), and poor/no response (score 3) (20). When both a numerical grade and narrative wording were present, the numerical grade was prioritized; this occurred in one case. The harmonization created an analytical ordinal variable and was not considered formal re-grading under Becker or another single system (7). MPR was defined as complete or near-complete response (scores 0-1), and pCR as complete response (score 0). Because residual viable-tumor percentages were not available for all patients, no MPR definition based on a common percentage threshold (e.g., <10% residual tumor) was applied.

Matching of response categories with pathology. Each patient’s endoscopic response category and, when available, CT response category were linked at the patient level to the harmonized pathological response of the same patient’s gastrectomy specimen. Endoscopic and CT categories were never converted into, or substituted for, pathological categories. The four-level endoscopic and CT categories were analyzed against the four-level pathological score as ordinal variables (Spearman correlation and cross-tabulation), against the binary MPR outcome as ordered predictors (ROC analysis), and, after collapsing to responder/non-responder, against MPR in 2×2 tables (sensitivity, specificity, PPV, NPV, accuracy).

### 2.4 Outcomes

The primary analysis compared the discrimination of endoscopy and CT for MPR in the 37-patient paired cohort. Secondary analyses evaluated the association between endoscopic response and pathological response in all 47 patients, binary performance measures for each modality, the apparent discrimination of an exploratory endoscopy-plus-CT model, the relationship between eCR and pCR, and MPR rates across combined binary response categories. Sensitivity analyses were restricted to cM0 patients who received FLOT alone and, separately, reclassified the single pathology report with discordant numerical and narrative descriptions.

### 2.5 Statistical analysis

No formal sample-size calculation was performed; all patients meeting the eligibility criteria were included. Continuous variables are summarized as mean (standard deviation) and median (range), and categorical variables as number (percentage). Spearman rank correlation was used to assess the association between ordered endoscopic or CT response and the four-level pathological response category.

For receiver operating characteristic analyses, endoscopic and CT response categories were ordered from complete response to progressive disease. Endoscopic performance was estimated in the full cohort and in the paired cohort. Sensitivity, specificity, positive predictive value (PPV), negative predictive value (NPV), and accuracy were calculated using the prespecified binary responder/non-responder definitions; 95% confidence intervals for proportions were calculated using the Wilson method. Paired endoscopy and CT AUCs were compared with the DeLong test (21).

An exploratory logistic regression model included the ordinal endoscopic and CT response variables. Because the model was developed and evaluated in the same 37 patients, its AUC represents an apparent, uncorrected estimate; calibration and clinical utility were not assessed. Fisher exact testing compared MPR prevalence between patients with and without comparable CT. Missing data were handled by available-case analysis. All tests were two-sided, with p<0.05 considered statistically significant. Analyses were performed using SPSS version 25.0 and Python.

## 3 Results

### 3.1 Cohort assembly and baseline characteristics

Of the 450 records screened, 403 were excluded (no FLOT-based therapy, n=10; no post-treatment endoscopy, n=254; insufficient endoscopy report to classify response, n=122; no tumor regression grade or pathological response documented in the pathology report, n=17), and 47 patients (10.4%) met the criteria for the endoscopy-pathology cohort. Of these, 37 had comparable paired CT assessments and 10 did not. All CT and combined-modality analyses were therefore limited to the paired cohort.

Median age was 64 years (range, 28-76), mean age was 61.1 years (SD, 11.4), and 28 patients (59.6%) were male. Tumors were located in the cardia/GEJ in 18 patients, corpus in 15, and antrum in 14. Most tumors were recorded as adenocarcinoma (42/47), 37 patients were classified as cT3, 35 were clinically node-positive, and 46 were cM0. Forty-five patients received FLOT alone, one received FLOT plus nivolumab, and one received FLOT plus another immunotherapy agent. Baseline characteristics are summarized in **Table 1**.

**Table 1:** Baseline clinical and treatment characteristics of the endoscopy-pathology cohort (n=47).

Characteristic	Value
Demographic characteristics	
Age, median (range), years	64 (28-76)
Age, mean (SD), years	61.1 (11.4)
Male sex	28 (59.6)
Tumor characteristics	
Primary location: cardia/GEJ	18 (38.3)
Primary location: corpus	15 (31.9)
Primary location: antrum	14 (29.8)
Recorded histology: adenocarcinoma, including mixed adenocarcinoma	42 (89.4)
Recorded histology: poorly cohesive/signet-ring carcinoma	3 (6.4)
Recorded histology: poorly differentiated carcinoma, NOS	1 (2.1)
Recorded histology: mixed neuroendocrine-non-neuroendocrine neoplasm	1 (2.1)
Recorded differentiation: well/moderate/poor or poorly cohesive/not stated	1/8/17/21 (2.1/17.0/36.2/44.7)
Clinical T category: cT1/cT2/cT3/cT4	1/4/37/5 (2.1/8.5/78.7/10.6)
Clinical N category: cN0/cN1/cN2/cN3	12/29/4/2 (25.5/61.7/8.5/4.3)
Clinical M category: cM0/cM1	46/1 (97.9/2.1)
Treatment and surgery	
Preoperative FLOT	45 (95.7)
FLOT plus nivolumab	1 (2.1)
FLOT plus other immunotherapy	1 (2.1)
Surgery: total/subtotal gastrectomy	27/20 (57.4/42.6)
Response assessments and biomarkers	
Endoscopic response: eCR/ePR/eSD/ePD	2/18/25/2 (4.3/38.3/53.2/4.3)
CT response in paired cohort (n=37): CR/PR/SD/PD	1/15/19/2 (2.7/40.5/51.4/5.4)
MMR/MSI status: MSS/MSI/not tested or reported	25/2/20 (53.2/4.3/42.6)
PD-L1 CPS available; median (range)	18 (38.3); 2 (0-80)
HER2 IHC available; score 3+	30 (63.8); 1/30 (3.3)
HER2 FISH available; amplified	26 (55.3); 4/26 (15.4)

Data are n (%) unless otherwise stated. Percentages are based on n=47, except for CT response (n=37) and biomarker-positive rates, which are calculated among tested patients. Abbreviations: CPS, combined positive score; CR, complete response; CT, computed tomography; eCR, endoscopic complete response; ePD, endoscopic progressive disease; ePR, endoscopic partial response; eSD, endoscopic stable disease; FISH, fluorescence *in situ* hybridization; FLOT, fluorouracil, leucovorin, oxaliplatin, and docetaxel; GEJ, gastroesophageal junction; HER2, human epidermal growth factor receptor 2; IHC, immunohistochemistry; MMR, mismatch repair; MSI, microsatellite instability; MSS, microsatellite stable; NOS, not otherwise specified; PD, progressive disease; PR, partial response; SD, stable disease.

### 3.2 Pathological response and full-cohort endoscopic performance

Pathological response was classified as complete in 10 patients (21.3%), near-complete in 7 (14.9%), partial in 15 (31.9%), and poor/no response in 15 (31.9%). MPR occurred in 17 of 47 patients (36.2%), including 10 patients with pCR.

Endoscopic response was eCR in 2 patients, ePR in 18, eSD in 25, and ePD in 2. The ordinal association between endoscopic and pathological response was weak and not statistically significant (Spearman rho=0.189, p=0.204; **Figure 2**). Binary endoscopic response was present in 9 of 17 patients with MPR and 11 of 30 patients without MPR. Sensitivity was 52.9%, specificity 63.3%, PPV 45.0%, NPV 70.4%, and accuracy 59.6% (**Table 2**). The endoscopic AUC in the full cohort was 0.619 (95% CI, 0.466-0.771).

**Figure 2:**
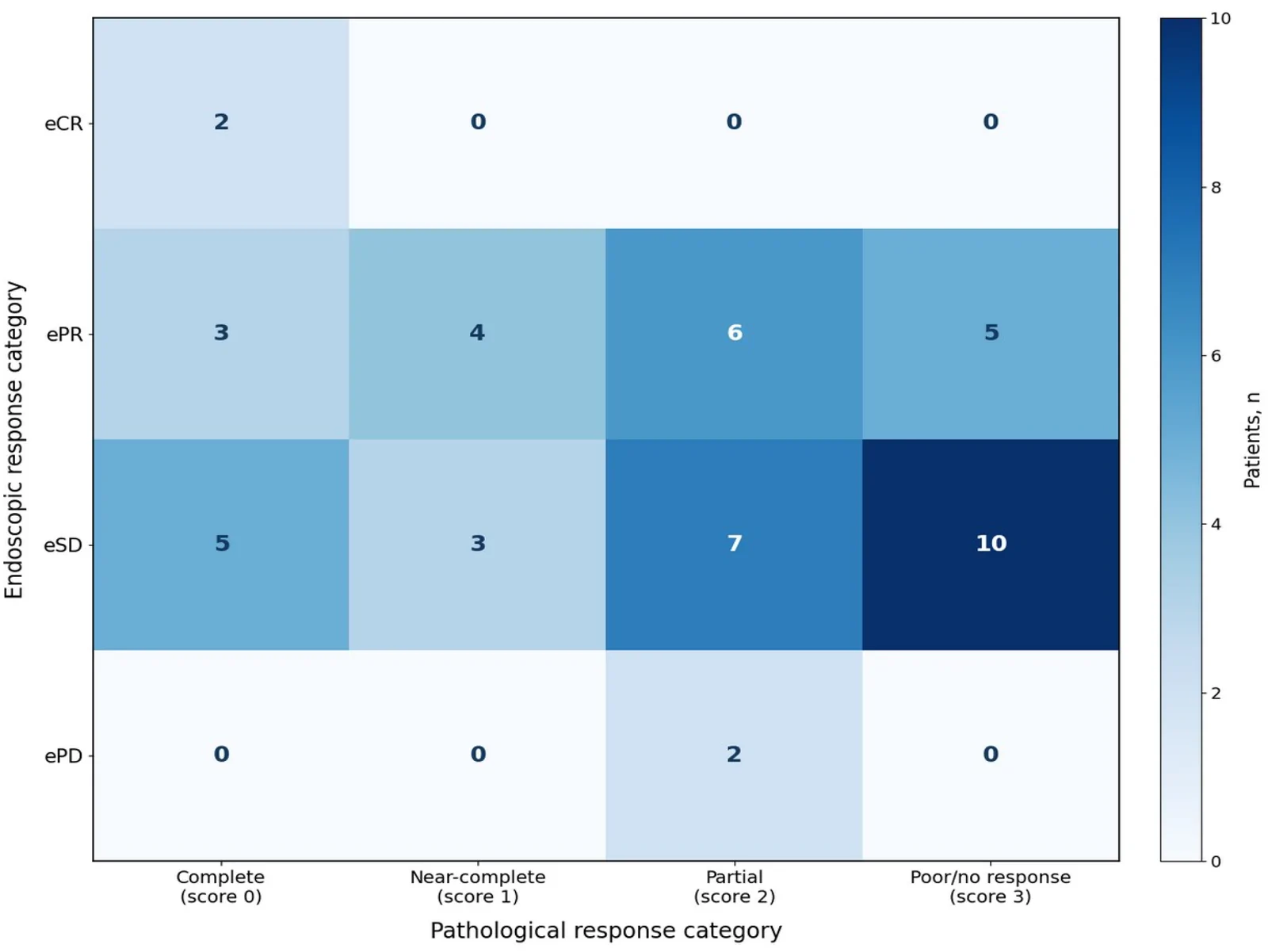
Distribution of endoscopic response across the four pathological response categories (n=47). Numbers within cells indicate patient counts.

**Table 2:** Performance of endoscopy and CT for major pathological response. Values are percentages (95% CI), except AUC.

Metric	Endoscopy, full cohort (n=47)	Endoscopy, paired cohort (n=37)	CT, paired cohort (n=37)
Sensitivity	52.9 (31.0-73.8)	54.5 (28.0-78.7)	63.6 (35.4-84.8)
Specificity	63.3 (45.5-78.1)	57.7 (38.9-74.5)	65.4 (46.2-80.6)
PPV	45.0 (25.8-65.8)	35.3 (17.3-58.7)	43.8 (23.1-66.8)
NPV	70.4 (51.5-84.1)	75.0 (53.1-88.8)	81.0 (60.0-92.3)
Accuracy	59.6 (45.3-72.4)	56.8 (40.9-71.3)	64.9 (48.8-78.2)
Ordinal AUC	0.619 (0.466-0.771)	0.561 (0.379-0.743)	0.675 (0.501-0.848)

AUCs are based on the ordered response categories; sensitivity, specificity, predictive values, and accuracy are based on the binary responder/non-responder definitions. In the paired cohort, endoscopic categories were limited to ePR and eSD. The paired AUC difference (CT minus endoscopy) was 0.114 (DeLong p=0.35). Abbreviations: AUC, area under the receiver operating characteristic curve; CI, confidence interval; CT, computed tomography; NPV, negative predictive value; PPV, positive predictive value.

Both patients classified as eCR had pCR. For identification of pCR, eCR had a sensitivity of 20.0% (2/10; 95% CI, 5.7%-51.0%), specificity of 100% (37/37; 95% CI, 90.6%-100%), and PPV of 100% (2/2; 95% CI, 34.2%-100%). These estimates should be interpreted cautiously because only two patients were classified as eCR.

### 3.3 Paired comparison of endoscopy and CT

The paired cohort included 37 patients, of whom 11 had MPR and 26 did not. Endoscopy had a sensitivity of 54.5%, specificity of 57.7%, PPV of 35.3%, NPV of 75.0%, and accuracy of 56.8%. Corresponding CT estimates were 63.6%, 65.4%, 43.8%, 81.0%, and 64.9%, respectively (**Table 2**). The ordinal association between CT response and pathological response was modest (Spearman rho=0.320, p=0.054).

The AUC was 0.561 (95% CI, 0.379-0.743) for endoscopy and 0.675 (95% CI, 0.501-0.848) for CT. The estimated difference of 0.114 in favor of CT was not statistically significant (paired DeLong p=0.35). The 95% CI for this difference (approximately -0.13 to 0.35) was wide and included both clinically meaningful superiority of CT and small differences in the opposite direction. The exploratory endoscopy-plus-CT model yielded an apparent AUC of 0.692 (95% CI, 0.511-0.873), an absolute increase of 0.017 over CT alone.

MPR occurred in 11 of 37 patients with comparable CT (29.7%) and 6 of 10 without comparable CT (60.0%; Fisher exact p=0.136). Notably, the paired cohort included only ePR and eSD observations; both eCR and both ePD observations occurred among the 10 patients without comparable CT. The paired endoscopic estimate therefore reflects a narrower response spectrum than the full endoscopy cohort.

### 3.4 Combined response categories

MPR occurred in 4 of 10 patients with both endoscopic and CT response, 2 of 7 with endoscopic response only, 3 of 6 with CT response only, and 2 of 14 with neither response (**Table 3**). The four groups did not show a monotonic response gradient. Although the dual non-response group had the lowest MPR rate, 2 of 14 patients in this group nevertheless had MPR.

**Table 3:** Major pathological response according to combined endoscopic and CT response (n=37).

Combined response category	Major response/n	Rate (%)
Endoscopy response/CT response	4/10	40.0
Endoscopy response/CT non-response	2/7	28.6
Endoscopy non-response/CT response	3/6	50.0
Endoscopy non-response/CT non-response	2/14	14.3

### 3.5 Sensitivity analyses

After restriction to cM0 patients treated with FLOT alone, 44 patients remained; 15 had MPR and 8 had pCR. The endoscopic AUC in the full restricted cohort was 0.633 (95% CI, 0.474-0.793). Among the 35 patients with comparable CT, AUCs were 0.600 (95% CI, 0.412-0.788) for endoscopy and 0.654 (95% CI, 0.468-0.840) for CT (paired p=0.662).

One pathology report combined a numerical Ryan grade 1 with a narrative description of partial response. The primary analysis prioritized the numerical score. Reclassifying this case as partial response reduced the MPR count from 17 to 16 but did not materially alter the results: the full-cohort endoscopic AUC was 0.597 (95% CI, 0.439-0.755), and paired-cohort AUCs were 0.528 (95% CI, 0.339-0.717) for endoscopy and 0.648 (95% CI, 0.463-0.833) for CT (p=0.361).

## 4 Discussion

In this retrospective cohort of patients treated predominantly with preoperative FLOT, routine endoscopic response was only weakly related to pathological response. In the paired analysis, CT produced a higher AUC point estimate than endoscopy, but the difference was not statistically significant and the confidence intervals were wide. Adding endoscopy to CT produced only a small apparent increase in AUC. Endoscopic complete response was associated with pCR in the two observed cases, but its sensitivity was low. Overall, routinely assigned endoscopic and CT response categories provided only modest information about the extent of primary-tumor regression.

Previous studies have generally reported more favorable associations between endoscopic response, histological regression, or survival (12–16). However, those studies differed from the present analysis in important respects, including chemotherapy regimen, assessment timing, response definitions, prospective scheduling, and the use of dedicated or expert review. The present study addresses a narrower real-world question: how response categories assigned from routine clinical reports relate to gastrectomy pathology in a contemporary, predominantly FLOT-treated cohort. The less favorable discrimination observed here may therefore reflect both the limitations of routine documentation and genuine biological discordance between modalities.

The observed pCR rate of 21.3% and MPR rate of 36.2% are broadly compatible with pathological regression reported in the FLOT era (22), although direct comparison is limited by cohort selection and differences in regression grading. The clinically relevant issue is not whether FLOT can induce substantial regression, but whether that regression can be recognized reliably before surgery. In this cohort, neither routine endoscopy nor routine CT achieved sufficiently precise discrimination to answer that question conclusively.

The modest concordance is biologically plausible. Endoscopy evaluates mucosal healing, ulceration, and luminal morphology, but these findings may not reflect residual viable tumor in deeper wall layers. CT can evaluate extraluminal disease and lymph nodes, yet RECIST response may be driven by target lesions other than the gastric primary. Pathological response, in contrast, is determined across the resected primary-tumor bed. A numerically higher CT AUC should therefore not be interpreted as evidence that CT is intrinsically superior for assessing primary-tumor biology. These results concern prediction of pathological regression and do not diminish the established, complementary roles of endoscopy and CT in clinical staging and reassessment.

The combined analyses also require cautious interpretation. MPR rates did not increase consistently across the four joint endoscopy/CT categories, and 2 of 14 patients with dual non-response nevertheless had MPR. The logistic model was developed and evaluated in the same small cohort and was not assessed for calibration or clinical utility. Similarly, the finding that both eCR patients had pCR is potentially informative but statistically fragile: eCR identified only 2 of 10 pCRs, and the confidence interval around its PPV was wide. eCR may merit prospective evaluation as a high-specificity signal, but it cannot be regarded as a stand-alone decision rule on the basis of these data.

The study also has several strengths. It focuses on a contemporary treatment setting, includes a within-patient comparison of endoscopy and CT, reports confidence intervals rather than relying only on point estimates, and presents sensitivity analyses addressing treatment heterogeneity and uncertainty in pathological coding. The transparent harmonization of pathology terminology also makes clear how the analytical endpoint was constructed.

Several limitations should be considered. Only 47 of 450 screened records met the documentation requirements for the endoscopy-pathology analysis; most exclusions were due to the absence of post-treatment endoscopy (n=254) or an endoscopy report insufficient to classify response (n=122). Selection according to the availability and completeness of paired endoscopy may have introduced selection and spectrum bias and limits generalizability. The large reduction from the screened population mainly reflects that restaging endoscopy is not performed uniformly before surgery in routine practice and that, when performed, reports are not always sufficiently detailed to classify response; the analytical cohort therefore represents patients in whom clinicians chose to obtain and document restaging endoscopy, and MPR prevalence and modality performance in the wider FLOT-treated population may differ. Because the decision to perform or to report restaging endoscopy in detail may itself be related to clinical characteristics, missing data cannot be assumed to be missing at random. CT was available in only 37 patients, with 11 MPR events, and the paired subset had a narrower endoscopic response spectrum and a lower MPR prevalence than the CT-unavailable group. Consequently, the comparative estimates were imprecise, a non-significant AUC comparison should not be interpreted as evidence of equivalence, and the combined-model AUC remains an unvalidated apparent estimate. With 11 MPR events, the paired comparison had sufficient power to detect only large differences in discrimination, and the primary comparison should be regarded as hypothesis-generating rather than confirmatory. Because characteristics of excluded records were not collected, the direction and magnitude of any selection bias cannot be quantified.

Response categories were derived from routine clinical reports without blinded central re-review, and the analysis dataset did not retain the original images, raw measurements, assessment timing, target-lesion definitions, or assessor characteristics. Interobserver reproducibility could not therefore be evaluated. In addition, pathological reports used heterogeneous grading systems and descriptive terminology, and the harmonized four-level scale may have introduced classification error around the MPR threshold. This risk is greatest for the 12 reports that used descriptive terminology alone and at the boundary between near-complete and partial response, which defines MPR; because residual viable-tumor percentages were not consistently available, a uniform percentage-based definition of MPR could not be applied, and the sensitivity analysis addressing the single report with discordant numerical and narrative descriptions cannot exclude misclassification in other reports. The cohort included one cM1 patient, two patients treated with immunotherapy plus FLOT, and one mixed neuroendocrine-non-neuroendocrine neoplasm; detailed treatment-intensity and timing variables were also unavailable. The sensitivity analyses were reassuring but cannot fully address these sources of heterogeneity, and the single-center design further limits external validity.

Future studies should use prospectively defined assessment intervals, standardized endoscopic criteria, primary-tumor-focused imaging measurements, blinded independent review, and uniform central pathological grading. Adequately powered development and external-validation cohorts will be needed to determine whether a multimodal approach can provide clinically useful preoperative estimates of pathological response. Until such evidence is available, endoscopy and CT should be interpreted as complementary assessments rather than substitutes for surgical pathology.

## 5 Conclusion

In patients undergoing gastrectomy after FLOT-based therapy, routine endoscopic response categories showed limited discrimination of MPR. CT was numerically more informative, but the paired comparison was inconclusive, and the combined model provided no established incremental value. Because the analytical cohort represented a selected minority of screened patients, the pathological endpoint was harmonized from heterogeneous reports, and the paired cohort was small, these findings should be regarded as hypothesis-generating. Standardized prospective multimodal assessment is needed to determine whether preoperative response can predict pathological regression reliably.

## Data Availability

The data supporting the findings of this study are not publicly available. Access to de-identified data may be considered by the corresponding author upon reasonable request and subject to institutional approval.
